# Differentiating Sporadic behavioural variant Frontotemporal Dementia from late‐onset Primary Psychiatric Disorders: the DIPPA‐FTD study

**DOI:** 10.1002/alz.087999

**Published:** 2025-01-03

**Authors:** Sterre C. M. de Boer, Chiara Fenoglio, Giorgio G Fumagalli, Lina Riedl, Sophie Matis, Zac Chatterton, Ishana Rue, Ramon Landin‐Romero, Sven J van der Lee, Patrick Sommer, Timo Grimmer, Janine Diehl‐Schmid, Charlotte Teunissen, Daniela Galimberti, Glenda M Halliday, Simon Ducharme, Olivier Piguet, Yolande A.L. Pijnenburg

**Affiliations:** ^1^ Amsterdam Neuroscience, Neurodegeneration, Amsterdam, Noord‐Holland Netherlands; ^2^ The University of Sydney, School of Psychology and Brain & Mind Centre, Sydney, NSW Australia; ^3^ Alzheimer Center Amsterdam, Neurology, Vrije Universiteit Amsterdam, Amsterdam UMC location VUmc, Amsterdam, Noord‐Holland Netherlands; ^4^ University of Milan, Fondazione Cà Granda, IRCCS Ospedale Policlinico, Milan Italy; ^5^ Department of Biomedical, Surgical and Dental Sciences. University of Milan, Milan Italy; ^6^ University of Trento, Rovereto, Italy, Rovereto Italy; ^7^ Technical University of Munich, School of Medicine, Munich Germany; ^8^ The University of Sydney, School of Health Sciences, Sydney, NSW Australia; ^9^ The University of Sydney, Brain & Mind Centre, Camperdown, NSW Australia; ^10^ Douglas Mental Health University Institute, McGill University, Montreal, QC Canada; ^11^ Alzheimer Center Amsterdam, Neurology, Vrije Universiteit Amsterdam, Amsterdam UMC location VUmc, Amsterdam Netherlands; ^12^ Amsterdam Neuroscience, Neurodegeneration, Amsterdam Netherlands; ^13^ Genomics of Neurodegenerative Diseases and Aging, Human Genetics, Vrije Universiteit Amsterdam, Amsterdam UMC, Amsterdam Netherlands; ^14^ Klinikum rechts der Isar, Technical University of Munich, School of Medicine, Munich Germany; ^15^ Technical University of Munich, München Germany; ^16^ Inn‐Salzach‐Klinikum, Wasserburg Germany; ^17^ Neurochemistry Laboratory, Department of Clinical Chemistry, Amsterdam Neuroscience, Vrije Universiteit Amsterdam, Amsterdam UMC, Amsterdam Netherlands; ^18^ Fondazione IRCCS Ca’ Granda, Ospedale Maggiore Policlinico, Milan Italy; ^19^ Department of Biomedical, Surgical and Dental Sciences, University of Milan, Milan Italy; ^20^ The University of Sydney, Sydney, NSW Australia; ^21^ Douglas Mental Health University Institute, Department of Psychiatry, McGill University, Montreal, QC Canada; ^22^ McConnell Brain Imaging Centre, Montreal Neurological Institute, McGill University, Montreal, QC Canada; ^23^ Alzheimer Center Amsterdam, Neurology, Vrije Universiteit Amsterdam, Amsterdam UMC, Amsterdam Netherlands

## Abstract

**Background:**

Sporadic bvFTD is often misdiagnosed as a primary psychiatric disorder (PPD) due to overlapping clinical features and lack of reliable biomarkers. The multi‐centre study DIPPA‐FTD aims to develop diagnostic‐ and prognostic*‐*algorithms that can distinguish sporadic bvFTD from late‐onset PPD. The aim of the retrospective DIPPA‐FTD study was to identify the strongest clinical discriminators.

**Method:**

DIPPA‐FTD has compiled a retrospective database with 508 sporadic bvFTD and 152 late‐onset PPD cases from five cohorts, making it the largest sporadic FTD cohort to date. Logistic regression models and ROC curve analysis were applied to determine discriminative value per clinical marker in separate subsets; (i) neuropsychological features, (ii) visual brain atrophy rating scales and (iii) serum NfL+GFAP. A global (backward stepwise) logistic regression was also conducted in the most optimal subset that had all markers per modality available. All models were adjusted for age, sex and education when indicated.

**Result:**

For marker (i) (bvFTD n = 217, PPD n = 75) higher scores of letter fluency (OR:1.47, p<0.001), global cognitive screening (OR:1.72, p = 0.01) and lower attention scores (OR:0.77, p = 0.05) were significantly associated with increased likelihood of PPD and reached an AUC of 0.77. Marker (ii) visual atrophy rating composite score (bvFTD n = 211, PPD n = 112) reached diagnostic accuracy of 79% and fronto‐insula was the most useful discriminator (AUC 0.80). Analysis of marker (iii) NfL+GFAP (bvFTD n = 275, PPD n = 82) showed that NfL and GFAP levels were significantly higher in bvFTD. Combination of NfL+GFAP yielded highest AUC value (0.88). The combined dataset with all markers variables available (bvFTD n = 120, PPD n = 40) reached an AUC of 0.89. Higher NfL (OR:1.09, p<0.01), more atrophy in fronto‐insula (OR:2.38, p = 0.02) and enlarged mean ventricular space (OR:3.84, p = 0.05) were significant predictors for sporadic bvFTD.

**Conclusion:**

Global cognition, letter fluency and attention scores, fronto‐insula brain atrophy and NfL+GFAP have a significant role in discriminating sporadic bvFTD from PPD. Combination of markers can increase diagnostic accuracy in clinical setting. Promising markers identified in this retrospective study will be validated in the prospective DIPPA‐FTD study and integrated in a data‐driven approach to develop diagnostic and prognostic tools, enabling early‐stage diagnosis sporadic bvFTD which is required for trial enrolment.

